# Development and validation of a post-operative delirium prediction model for patients undergoing abdominal surgery: a retrospective, observational, single-center study

**DOI:** 10.1186/s13741-025-00639-0

**Published:** 2026-02-28

**Authors:** Zhi-Hua Huang, Maneesh Kumarsing Beeharry, Xiao-Ying Xu, Cheng-Rong Bao, Lei Tao, Yan Luo

**Affiliations:** 1 Department of Anaesthesiology, Ruijin Hospital Affiliated to Shanghai Jiao Tong University School of Medicine, 197 Ruijin Er Road, Huang Pu District, Shanghai, 200025 China; 2 Department of Surgery, Ruijin Hospital Affiliated to Shanghai Jiao Tong University School of Medicine, Shanghai, China

**Keywords:** Postoperative delirium, Abdominal surgery, Prediction model, Anaesthesia, Nomogram

## Abstract

**Background:**

Postoperative delirium (POD) is a common complication following major surgery in elderly. The purpose of this study was to develop and evaluate a POD prediction model for patients undergoing abdominal surgery.

**Methods:**

One thousand consecutive patients scheduled for elective abdominal surgery from July 2019 to March 2021 in Ruijin Hospital, Shanghai China, were retrospectively analysed, and their demographics, pre-operative evaluation, and intra-operative parameters were collected and cross-analysed. The primary outcome was the POD incidence. A prediction model of POD was established and internal validation was conducted with various analyses including univariate and multivariate regression. Data from another cohort of346 patients enrolled from July 2021 to December 2021 were used for model external validation.

**Results:**

After screening, 838 patients were included as the training cohort and 10.9% (91/838) of the patients manifested POD. Old age, cerebrovascular disease and diazepam use history and intraoperative fluid imbalance were the main contributors of the POD prediction model. The optimum cut-off point of the predicted probability that maximised the sum of sensitivity and specificity was 0.12. The fitting set AUC was 0.703 (95% Confidence interval (CI) 0.637–0.753). The sensitivity and specificity of the model were 0.556 and 0.754 respectively. The mean AUC during the cross and external validation of the model was 0.684 [Standard Deviation (SD) 0.068] and 0.634 (95%CI 0.511–0.758) respectively.

**Conclusions:**

Our data indicated that improving perioperative management may reduce POD incidence in patients who are old age and have cerebrovascular disease history.

**Trial registration:**

The retrospective data (ChiCTR2100047405) of this study was registered in the Chinese Clinical Trial registry (https://www.chictr.org.cn/).

## Introduction

Postoperative delirium (POD) is a common complication in elderly following major abdominal surgery and poses adverse impacts on postoperative recovery including triggering cognitive impairment and even dementia development, decreased functional independence, and increasing family and social financial burdens, as well as heightened mortality (McDonald et al. 2018; Chen et al. 2017; Goldberg et al. 2020; Sadlonova et al. 2022; Janssen et al. 2019). The incidence of POD after major surgical procedures can be up to 61% and POD is typically manifested during the first 7 postoperative days as previously reported (Ahrens et al. 2023; Jiang et al. 2023; Ackenbom et al. 2023; Li et al. 2022). Unfortunately, once an initial POD episode occurs, post-episode treatment or intervention has little effect on severity, duration, or likelihood of recurrence (Burry et al. 2018; Friedman et al. 2014). However, POD is preventable in 30–40% of cases and therefore, early identification of high-risk patients is of importance for early implement of effective prevention and management (Inouye et al. 2014; Deeken et al. 2022; Humeidan et al. 2021).

In order to prevent POD and optimize its outcomes, it is henceforth critical to identify preoperatively the high-risk factors for POD (Staveski et al. 2021; Hughes et al. 2020). Several risk factors associated with POD, such as advanced age, dementia or psychiatric comorbidity, cognitive deficiencies, uncertain polypharmacy, postoperative electrolyte and metabolic disorders and cerebrovascular events were previously reported (Huang et al. 2021, 2019; Zhou et al. 2021; Hiraki et al. 2021; Jong et al. 2019). Nevertheless, advanced age has been the most commonly reported independent risk factor for POD. In the past, there were numerous attempts to build POD prediction models based on the related high-risk factors. However, due to insufficient clinical data, the POD prediction models built up based on these factors is not yet optimised for routine clinical use. Therefore, in this study, the preoperative, intraoperative and postoperative factors associated with the incidence of POD after abdominal surgery were analysed to develop and validate a POD prediction model.

## Material and methods

### Ethics approval

This study was approved by the Ethics Committee of Ruijin Hospital affiliated to Shanghai Jiao Tong University School of Medicine, Shanghai, China and then registered in the Chinese Clinical Trial Registry Platform (WHO-ICTRP) (ChiCTR2100047405).

### Patients

**Fig. 1:**
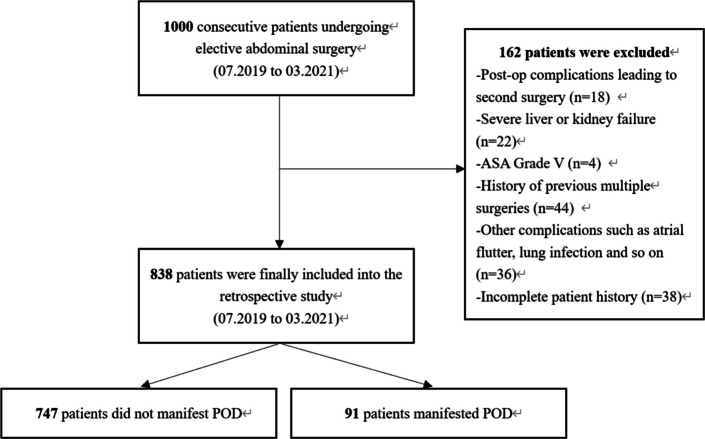
Study flowchart

In this study, 1000 consecutive patients scheduled for elective abdominal surgery in Ruijin Hospital affiliated to Shanghai Jiao Tong University School of Medicine from July 2019 to March 2021 were screened for eligibility. A final total of 838 patients were included and 162 patients were excluded due to various reasons (Fig. 1). Of those of 838, data from 586 patients were retrospectively analysed to establish a POD prediction model and data from 252 were used for internal validation the model. Data from another cohort of 346 patients prospectively enrolled l between July 2021 and December 2021 were used for external of the proposed model. The inclusion criteria were: (1) Age ≥ 18 years old; (2) ASA I-IV; (3) Underwent elective surgery; (4) No drug allergy. The exclusion criteria were: (1) Critical condition (ASA grade V); (2) Severe liver and kidney disease; (3) Unplanned secondary surgery due to post-operation complication; (4) Underwent general anaesthesia and surgery within three months; (5) Preoperative delirium.

### Data collection

Demographics, preoperative and intraoperative data were collected. Demographics included age, gender, body mass index (BMI), level of literacy, alcohol consumption (estimated weekly intake ≥ 200 mL of alcohol), smoking history (at least 10 cigarettes a day for the past 3 years), sleep quality, diazepam use, psychiatric disorders, and cerebrovascular disease history, and anxiety scores. Pre-operative data included American Society of Anaesthesiologists (ASA) physical status classification, blood laboratory measurements (albumin, haemoglobin, blood glucose, total bilirubin, urea, creatinine, sodium level, phosphorus level and white blood cell). Intraoperative data included surgery site, surgery duration, dose of anaesthetics, muscle relaxants and opioids fluid infusion, blood transfusion, blood loss, and urine output. Postoperative data including intensive care unit (ICU) admission was also collected.

### Diagnosis of POD

POD was diagnosed according to the Confusion Assessment Method (CAM) (Inouye et al. 1990) as routine clinical practice in our hospital. The assessment for POD was performed twice per day until postoperative day 7.

### Statistical analysis

Continuous variables were presented as mean ± standard deviation (SD) and categorical variables were expressed as number (%). For continuous data, unpaired* t* tests were used to compare the difference of two groups, and for categorical factors. Chi-square test was used. Factors with *P*-value less than 0.05 were used for univariate analysis and further analysed with multivariate logistic regression to establish a POD prediction model. The point estimates and 95% CIs for the adjusted odds ratios of independent risk factors, as well as the adjusted *P*-values of covariate coefficients, were provided through the multivariate logistic regression model. The cut-off point of the predicted values was selected to maximize the sum of sensitivity and specificity. In addition, in order to evaluate the prediction performance of the constructed model, the cross-validation were carried out to correct the bias during training phase, The external validation was conducted and a nomogram based on the determinants identified in the multivariate logistic model was established using rms package in R Project v.3.6.1. The model discrimination performance was evaluated by AUC, and the model calibration is evaluated by calibration curve. All the analyses were performed by R 3.6.1. (The R Foundation for Statistical Computing, Vienna, Austria. http://www.r-project.org) and Statistical Product and Service Solutions (SPSS) 19.0 (IBM, Armonk, NY, USA) software whereby statistical difference was defined by *P* < 0.05 (2-sided).

## Results

### Description of study population

Of the 838 participants, 10.9% (91/838) were diagnosed with POD. The incidence of POD was typically observed on day 2 after surgery (range, 1–3 days) and more than 95% of the patients were diagnosed within 3 days. All 91 patients were relieved of POD, with the mean duration of the POD of 3 days. The patients’ demographics between two groups (POD vs Non-POD) are listed in Table 1.

**Table 1:** Patients’ demographics parameters

Parameters	No POD (*n* = 747)	POD (*n* = 91)	*P* Value
Sex (%)			0.27
Male	319 (42.7)	45 (49.5)	
Female	428 (57.3)	46 (50.5)	
Age (mean ± SD, years)	58.7 ± 12.9	63.8 ± 11.7	< 0.001
BMI	22.8 ± 3.26	23.2 ± 3.35	0.21
Level of literacy (%)			0.37
< 6 years of schooling	187 (25.0)	29 (31.9)	
< 12 years of schooling	383 (51.3)	43 (47.3)	
≥ 12 years of schooling	177 (23.7)	19 (20.9)	
Alcohol consumption (%)	112 (15.0)	8 (8.8)	0.15
Smoking (%)	128 (17.1)	11 (12.1)	0.28
Sleep disorder (%)	193 (25.8)	27 (29.7)	0.51
History of diazepam use (%)	19 (2.5)	9 (9.9)	< 0.001
History of psychiatric disorders (%)	20 (2.7)	7 (7.7)	0.03
History of cerebrovascular disease (%)	68 (9.1)	20 (22.0)	< 0.001

### Patient demographics

Three hundred sixty-four patients were males (43.4%) while 474 were females (56.6%). The mean age was 59.3 ± 12.8 years (range, 18–86 years). The majority of patients had no history of alcohol or smoking, and there were no significant differences in sex, BMI, sleep disorders, and schooling years between the 2 groups. Notably, postoperative delirium patients were older (*p* < 0.001) and had a higher proportion of history of diazepam use (*p* < 0.001), psychiatric disorders (*p* = 0.03) and cerebrovascular disease (*p* < 0.001).

### Preoperative parameters

There were no significant differences in the ASA score and the preoperative blood parameters such as hemoglobin, white blood cell, albumin, glucose, total bilirubin, BUN, creatine, sodium and phosphorus with relevance to the occurrence of POD.

### Intraoperative parameters

Of the 706/838 (84.2%) patients who underwent upper abdomen surgery, 84/706 (11.9%) patients developed POD. On the other hand, of the 132/838 (15.8%) who underwent lower abdomen surgery, only 7/132 (5.3%) patients developed POD. The difference was statistically significant (*p* = 0.04). The operation time was longer than 3 h in 554/838 (66.1%) patients, and the difference was significant between the POD group (73, 80.2%) and non-POD group (481, 64.4%) (*p* = 0.04). Subsequently, the patients who developed POD had higher dosage of intra-operative sufentanyl (*p* = 0.01), higher fluid intake (*p* < 0.001) and positive fluid balance (*p* < 0.001). When analysing the association between intraoperative blood perfusion and POD incidence, it was noticed that 52.7% of the POD patients and 38.0% of the non-POD patients received intra-operative blood perfusion (*P* = 0.01).

### External validation population

Another pool of 346 patients undergoing abdominal surgery was used for the external validation of the model. There were 194 males (56.1%) and 152 (43.9%) females with a mean age of 64.37 ± 12.08 years (range, 19–87 years). Of the 346 participants as external validation set, 6.65% (23/346) were diagnosed with POD.

### Risk factors of POD

The univariate analysis showed that the following 11 factors showed statistical difference: age, history of diazepam use, history of cerebrovascular disease, history of psychiatric disorders, preoperative blood glucose, surgery site, surgery duration, intra-operative sufentanyl, total intraoperative fluid infusion, blood transfusion and fluid balance (*P* < 0.05). The statistically significant factors in univariate analysis were included as independent variables, and POD was included as the dependent variable in the multivariate logistic regression analysis. Since the intraoperative fluid balance and total intraoperative fluid infusion are strongly correlated with a correlation of 0.883 (*P* value < 10^–12^), based on previous literature, only fluid balance was included in the model (Smulter et al. 2018; Mailhot et al. 2019; Nguyen et al. 2018). By multivariate logistic regression analysis, 4 independent risk factors for POD development were obtained: age (OR = 1.280, *P* = 0.025), history of diazepam use (OR = 3.386, *P* = 0.007), history of cerebrovascular disease (OR = 1.978, *P* = 0.032) and the intraoperative fluid balance (OR = 1.269, *P* = 0.045) (Tables 2 and 3).

**Table 2:** Patients’ preoperative parameters

Parameters	No POD (*n* = 747)	POD (*n* = 91)	*P* Value
ASA score (%)			0.69
1 (I-II)	656 (87.8)	78 (85.7)	
2 (III-IV)	91 (12.2)	13 (14.3)	
Albumin level (%), g/L			0.36
Normal (≥ 35)	654 (87.6)	76 (83.5)	
Low (< 35)	93 (12.4)	15 (16.5)	
Hemoglobin (%), g/L			0.59
Normal (≥ 120)	494 (66.1)	57 (62.6)	
Low (< 120)	253 (33.9)	34 (37.4)	
Blood glucose (mean ± SD), mmol/L	5.8 ± 2.0	6.5 ± 2.9	0.03
Total bilirubilin (mean ± SD), umol/L	22.6 ± 39.0	28.8 ± 49.7	0.25
Urea (mean ± SD), mmol/L	5.5 ± 8.21	5.1 ± 1.63	0.19
Creatinine (mean ± SD), umol/L	73.7 ± 18.7	73.7 ± 17.8	0.99
Sodium (mean ± SD), mmol/L	141 ± 6.5	141 ± 2.9	0.41
Phosphorus (mean ± SD), mmol/L	1.1 ± 0.2	1.1 ± 0.2	0.68
White blood cells (mean ± SD), × 109/L)	6.0 ± 3.3	5.6 ± 1.7	0.05

**Table 3:** Patients’ intraoperative parameters

Parameters	No POD (*n* = 747)	POD (*n* = 91)	*P* Value
Surgery site (%)			0.04
Upper abdomen	622 (83.3)	84 (92.3)	
Lower abdomen	125 (16.7)	7 (7.7)	
ICU admission (%)	263 (35.2)	42 (46.2)	0.05
Surgery duration (%)			0.004
< 3 h	266 (35.6)	18 (19.8)	
≥ 3 h	481 (64.4)	73 (80.2)	
Rocuronium bromide (mean ± SD), mg/kg	1.2 ± 0.5	1.3 ± 0.6	0.08
Sufentanyl (mean ± SD), ug/kg	0. 9 ± 0.2	1.0 ± 0.3	0.01
Remifentanyl (mean ± SD), mg/kg	0. 02 ± 0.03	0.02 ± 0.01	0.96
Fluid infusion (mean ± SD), ml	3490 ± 1570	4040 ± 1300	< 0.001
Blood transfusion (%)	284 (38.0)	48 (52.7)	0.01
Blood loss (median [IQR]), ml	200 [5, 8000]	200 [20, 1600]	0.33
Urine output (mean ± SD), ml	600 ± 445	695 ± 464	0.07
Fluid balance (mean ± SD), ml	2570 ± 989	2970 ± 1060	< 0.001

### Development and validation of the POD prediction model

**Fig. 2:**
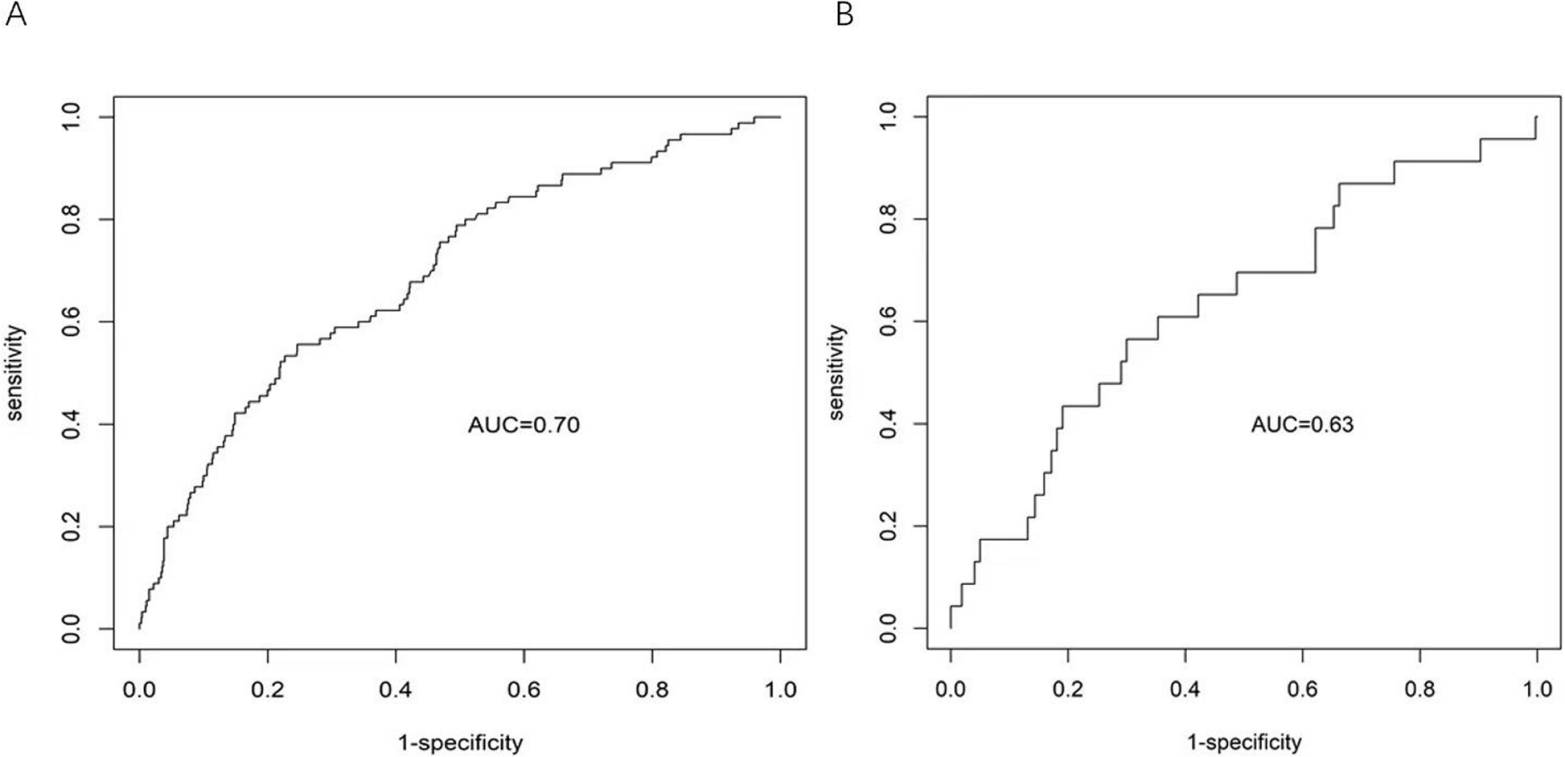
AUC and calibration curve of the 4-factor POD prediction model. **A** AUC of the fitting set. **B** AUC of the external validation set

The 4 factors were used to build our POD prediction model (Table 4). The AUC based on the prediction of the fitting set was 0.703 (95%CI: 0.637–0.753), indicating a good prediction effect. The optimum cut-off point of the predicted probability that maximized the sum of sensitivity and specificity was 0.12. The mean cross-validation AUC of the selected model was 0.684 (SD = 0.068), and the external validation AUC of the selected model was 0.634 (95%CI: 0.511- 0.758) and quite closed to the AUC based on the fitting set (0.703). Moreover, the model’s calibration evaluated by the fitting set calibration curve also indicated that the selected model was robust and performed well in prediction (Fig. 2).

**Table 4:** Multivariate logistic analysis of factors associated with POD

Factor	*p* Value	OR	95% CI
Age	0.025	1.280	1.032–1.587
History of diazepam use	0.007	3.386	1.395–8.222
History of psychiatric disorders	0.207	1.860	0.710–4.873
History of cerebrovascular disease	0.032	1.978	1.062–3.684
Preoperative blood glucose	0.132	1.446	0.895–2.335
Surgery site	0.145	1.835	0.810–4.156
Surgery duration	0.161	1.559	0.838–2.899
Intra-operative use of sufentanil	0.587	1.325	0.480–3.657
Fluid balance	0.045	1.269	1.005–1.603
Blood transfusion	0.920	1.028	0.597–1.771

**Fig. 3:**
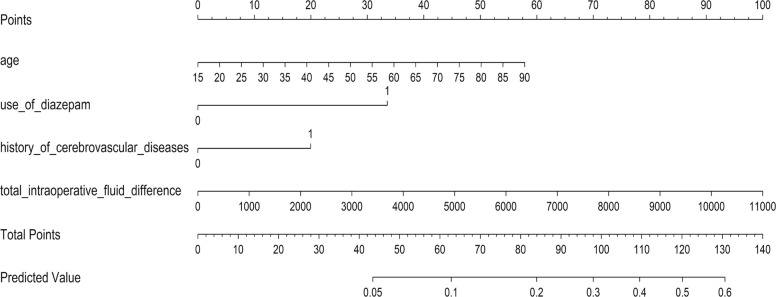
Nomogram of the POD prediction model for patients undergoing abdominal surgery. History of diazepam use: 0 = no, 1 = yes. History of cerebrovascular diseases: 0 = no, 1 = yes

By the constructed prediction model, the predicted probability of a patients experiencing POD is exp (h)/(1 + exp(h)), where h = −5.052 + 0.296 * (age/10) + 1.287 * (history of diazepam use) + 0.765* (history of cerebrovascular disease) + 0.349*(fluid balance/1000). Here value of categorical variables took 1 if history of diazepam use or history of cerebrovascular disease was true and 0 otherwise. The nomogram of the prediction model was given in Fig. 3.

## Discussion

Our study demonstrated that old age, cerebrovascular disease, diazepam use history and intraoperative fluid imbalance were the main contributors to the POD development. POD is a common complication following major surgeries and is associated with considerable postoperative poor outcomes. In our study, we reported a POD incidence rate of 10.9% (91/838) after elective abdominal surgeries, which is slightly lower than previous literature such as Gong et al. (2023), reported a POD incidence rate of 13.9% in a cohort of 1148 Chinese patients undergoing abdominal surgery. Given the established association between POD and elevated post-operative morbidity and mortality, especially in context of cognitive decline, identifying risk factors related to POD during the preoperative assessment is of utmost importance. Such identification can instigate the formulation of prophylactic strategies aimed to reduce its incidence with the ultimate goal of improving patient outcomes.

The pathogenesis of POD is thought to result from the interaction the predisposing and precipitating factors (Wittmann et al. 2022; Eschweiler et al. 2021). Common predisposing risk factors include advanced age, pre-existing cognitive deficits, prior dementia, psychological or emotional disruptions, and specific comorbidities such as cerebrovascular disease and alcohol abuse. Higher ASA physical status, non-elective surgery and postoperative pain had also been identified as precipitating factors for POD (Kim et al. 2020; Wang et al. 2019; Ziman et al. 2020; Lee and Sung 2020; Jin et al. 2020; O’Gara et al. 2021; Tang et al. 2020; Culley et al. 2017). Diabetes Mellitus (DM) stands out as a prevalent comorbidity or metabolic disorders associated with POD, with the underlying mechanism often attributed to polyneuropathy in advanced DM. However, further research is required to clarify the precise relationship between DM and POD (Xing et al. 2019; Chaiwat et al. 2019).

In this study, we found that patients prone to POD were more likely to be over 60 years, have a history of diazepam use and cerebrovascular disease and were exhibit positive higher intraoperative fluid balance. These findings are consistent with previous studies (Ziman et al. 2020; Lee and Sung 2020; Jin et al. 2020; O’Gara et al. 2021; Tang et al. 2020; Culley et al. 2017; Xing et al. 2019; Chaiwat et al. 2019; Ida et al. 2020), which have similarly implicated age, diazepam use, and cerebrovascular disease as risk factors for POD. However, in our cohort, no preoperative laboratory indicators were found to be statistically significant predictors of POD. Notably, we observed that positive intraoperative fluid balance was a significant contributor to POD. This aligns with findings from prediction models for cardiopulmonary and neurosurgeries, where intraoperative fluid imbalance has been shown to have both clinical and statistical significance (Lima et al. 2019; Seo et al. 2014; Viderman et al. 2020). Nagae et al. demonstrated that intravenous isotonic and hypotonic maintenance fluid were associated with an increase of POD in adult postoperative patients (Nagae et al. 2019). We speculate that positive fluid balance during surgery may lead to fluid overload, which can affect haemoglobin and haematocrit levels, as well as electrolyte imbalance. Smulter et al. reported that fluid overload increasing interstitial space and edema formation might interfere brain function, potentially contributing to the development of POD (Smulter et al. 2018). Interestingly, a history use of diazepam has been reported to cause dementia in elderly (Islam et al. 2016; Devlin et al. 2018), but yet it has not been incorporated in any prediction model up to date. Among the 91 patients who developed POD, 9 (9.9%) had a history of diazepam use, whereas among the 747 non-POD patients, only 19 (2.5%) had used diazepam previously. Thus, diazepam use is indeed a significant factor contributing to POD, but the underlying mechanism remains to be further explored.

In previously reported POD prediction models, advanced age, a prior history of cognitive deficiencies, along with functional and sensory impairments have been the most prevalent variables. Nevertheless, the absence of uniformity and standardization in the definition of these variables led to discrepancies in the application and comparison of these prediction models. To mitigate such variances, some POD prediction model excluded cognitive variables and instead incorporated hearing impairment as a predictive factor (Visser et al. 2021; Sanson et al. 2018). However, cognitive deficiency was reported to be one of the most common underlying causes for delirium development (Huang et al. 2021; Duning et al. 2021; Kang et al. 2020). Unfortunately, the absence of precise and reliable tools to evaluate subtle cognitive decline limits the appropriate inclusion of cognitive deficiency in prediction model. Some previous models have employed dementia evaluation criteria, yet for POD, these criteria was found to be insufficiently precise and accurate. In our model, we prioritised clinical factors, focusing on detailed medical history and medication use.

A robust clinical model is based on its predictive efficacy and clinical feasibility. In this study, we distilled the findings of the multivariate analysis and clinical implications to develop a POD prediction model for abdominal surgery patients. The model incorporates four predictors: age, diazepam use history, cerebrovascular disease history and fluid balance. Receiver operating characteristic curve (ROC) analysis yielded an area under the ROC curve (AUROC) of 0.703, indicating a commendable prediction effect. Although the model’s sensitivity was modest, the selected cut-off point was carefully determined to balance sensitivity and specificity for comprehensive risk assessment. This value reflects the inherent trade—off; a higher cut—off would boost specificity but compromise sensitivity. Future efforts should focus on optimising the model to improve sensitivity without sacrificing too much specificity. Additionally, exploring alternative cut-off points could further refine the model to meet diverse clinical requirements. Despite the relatively subdued sensitivity, the model demonstrated reasonable discriminative power with an AUC of 0.703 in the training set. This implies its utility in identifying patients at elevated POD risk. However, the sensitivity of 0.556 indicates substantial room for improvement, particularly in detecting POD within high-risk subgroups. Future research will focus on integrate additional risk factors and refine the model, with the goal of improving both sensitivity and overall predictive accuracy.

Nevertheless, this study has some limitations. First, the single-center nature of the study restricted its generalizability of our findings. Second, it is the retrospective nature and this underscores the necessity for further refinement to more accurately identify patients at risk of POD. Thirdly, long term follow up data are not available and, hence, how thses factors and their relations to POD contribute to long term outcomes is unknown. Lastly, data reported here were derived from single ethnic Chinese and, therefore, the generosity of our work is unknown.

## Conclusion

The 4-factor POD prediction model (age, diazepam use history, cerebrovascular disease history and fluid balance) shows good prediction efficiency and better perioperative management and even prophylactic intervention should be applied to patients who are at a high risk of POD.

## Data Availability

No datasets were generated or analysed during the current study.

## Abbreviations

POD: Postoperative Delirium
OR: Odds Ratio
CI: Confidence Interval
AUC: Area under the Curve
SD: Standard Deviation
WHO-ICTRP: World Health Organization International Clinical Trials Registry Platform
ASA: American Society of Anesthesiologists
BMI: Body Mass Index
ICU: Intensive Care Unit
CAM: Confusion Assessment Method
AIC: Akaike Information Criterion
DM: Diabetes Mellitus
